# Supplementary material for: Synthesis-free PET imaging of brown adipose tissue and TSPO via combination of disulfiram and 64CuCl2
Source: Sci Rep. 2017 Aug 15;7:8298. doi: 10.1038/s41598-017-09018-2 (PMC5557754; doi:10.1038/s41598-017-09018-2)
Supplement: Supplementary file 1 — Supplementary Information [file 41598_2017_9018_MOESM1_ESM.pdf]

Synthesis-free PET imaging of brown adipose tissue and TSPO via combination of disulfiram and $^{64}CuCl_2$

Jing Yang[1,2,5], Jian Yang[1,3,5], Lu Wang[4], Anna Moore[1], Steven H. Liang[4], and Chongzhao Ran[1]*

[1]Molecular Imaging Laboratory, Athinoula A. Martinos Center for Biomedical Imaging, Massachusetts General Hospital/Harvard Medical School, Boston, MA, 01890;

[2]College of Pharmaceutical Sciences, Soochow University, Suzhou, 215006, China.

[3]School of Pharmacy, China Pharmaceutical University, Nanjing, 210009;

[4]Division of Nuclear Medicine and Molecular Imaging & Gordon Center for Medical Imaging, Massachusetts General Hospital/Harvard Medical School, Boston, Massachusetts 02114.

[5] These authors contributed equally to this work.

Corresponding authors: Chongzhao Ran, cran@nmr.mgh.harvard.edu

Supplemental Figures

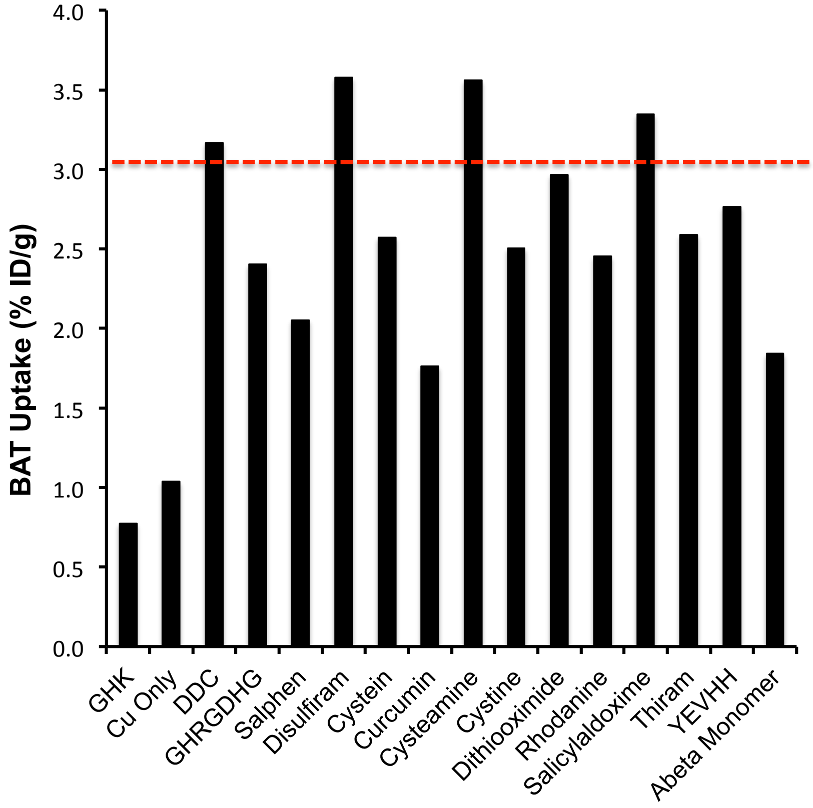

SI Fig.1 Top-down screening of Copper (II) ligands for BAT imaging.

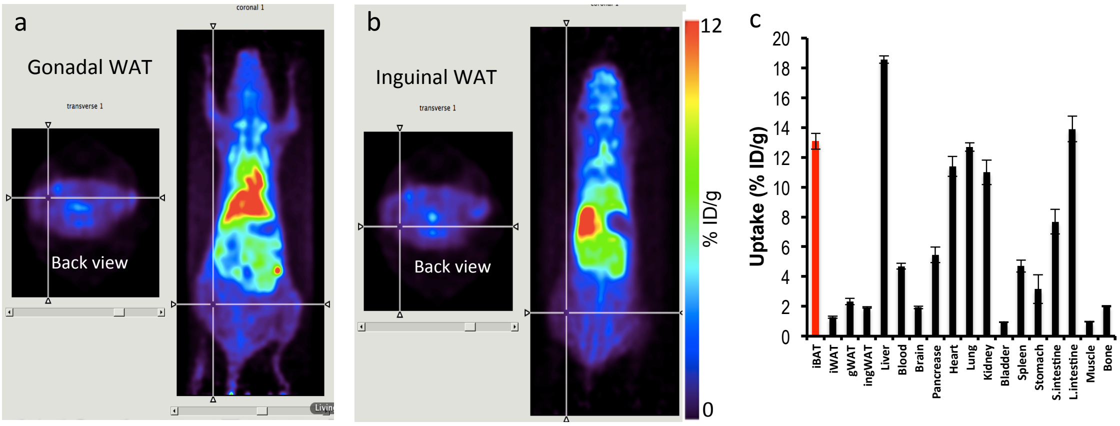

SI Fig.2 a-b) Representative images of ^{64}Cu-Dis uptake in gonadal and inguinal WAT. c) Full ex vivo bio-distribution data with ^{64}Cu-Dis.

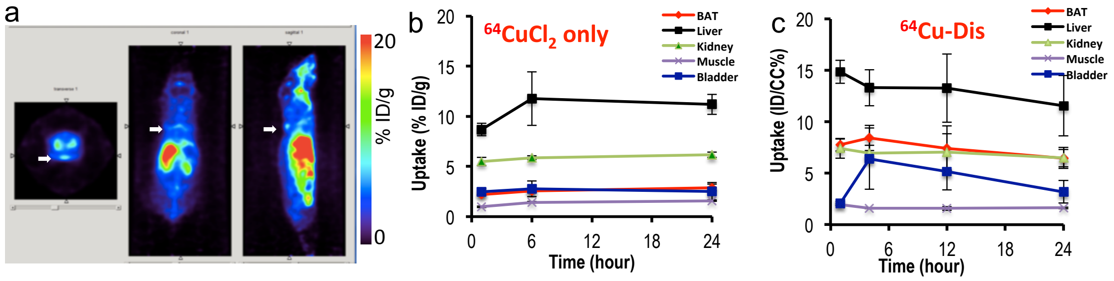

SI Fig.3 a) Representative BAT images with $^{64}CuCl_2$ only 1 hour after i.v. injection. b) Time activity curves of BAT, liver, kidney, bladder, and muscle after the injection of $^{64}CuCl_2$ only. c) Time activity curves of BAT, liver, kidney, bladder, and muscle with the step-wise protocol of ^{64}Cu-Dis.

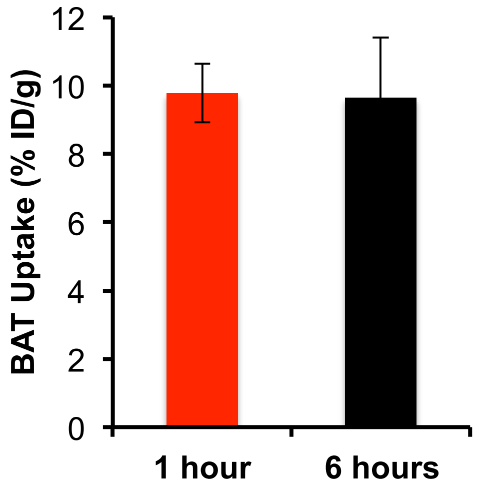

SI Fig.4 BAT uptake at 1 hour and 6 hours post ^{64}Cu-Dis injection.

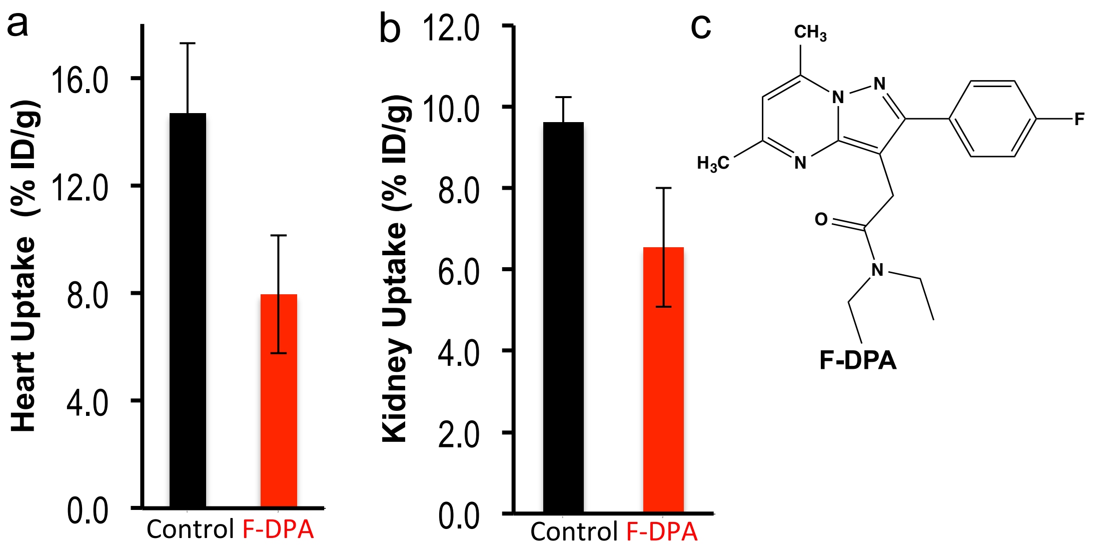

SI Fig.5 Quantitative analysis of heart (a) and kidney (b) images with F-DPA blocking at 1-hour post $^{64}CuCl_2$ injection. (c) Chemical structure of F-DPA.

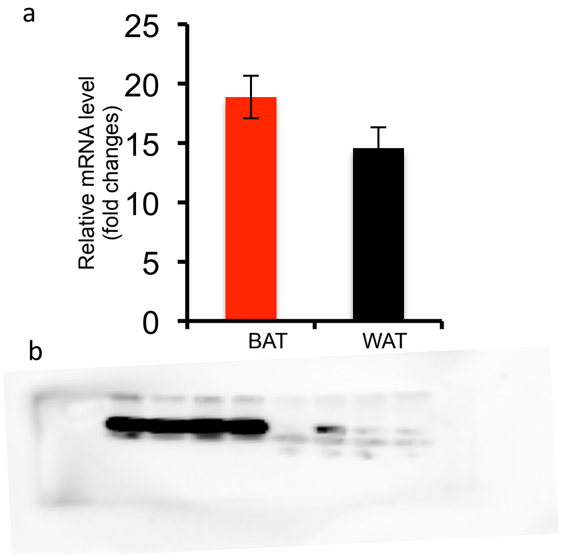

SI Fig.6 a) Quantitative PCR analysis of TSPO mRNA level in BAT and WAT tissues. b) Full length of western blot of Fig.4a.

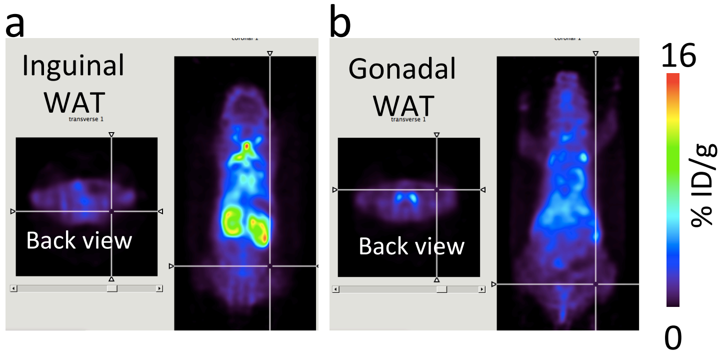

SI Fig.7 Representative images of [^{18}F]-F-DPA uptake in gondal and inguinal WAT.

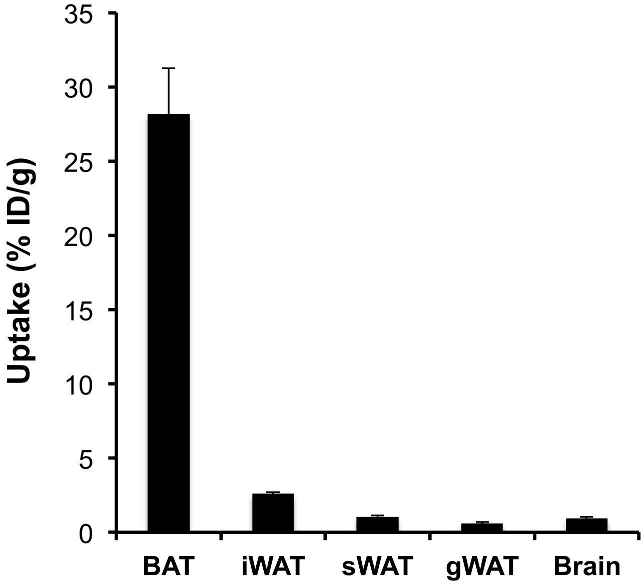

SI Fig.8 Ex vivo bio-distribution analysis of [^{18}F]-F-DPA uptake in BAT and WAT.
